# Sex‐specific risk profiles for substance use among college students

**DOI:** 10.1002/brb3.1959

**Published:** 2020-11-21

**Authors:** Caitlin E. Martin, Albert J. Ksinan, Frederick Gerard Moeller, Danielle Dick, Spit for Science Working Group

**Affiliations:** ^1^ Department of Obstetrics and Gynecology and VCU Institute for Drug and Alcohol Studies Virginia Commonwealth University School of Medicine Richmond VA USA; ^2^ Department of Health Behavior and Policy Virginia Commonwealth University Richmond VA USA; ^3^ Wright Center for Clinical and Translational Research and VCU Institute for Drug and Alcohol Studies Virginia Commonwealth University Richmond VA USA; ^4^ Developmental Psychology Program Department of Psychology and Human & Molecular Genetics Virginia Commonwealth University Richmond VA USA

**Keywords:** addiction, gender, sex, students, substance use disorder, young adults

## Abstract

**Introduction:**

Growing evidence indicates sex and gender differences exist in substance use. Framed by a lifecourse perspective, we explored prospectively by sex the effects of distal and proximal factors on the initiation of drug use in college.

**Methods:**

College students without prior drug use (*n* = 5,120 females; *n* = 2,951 males) were followed longitudinally across 4 years. Analyses were estimated as a multigroup survival analysis separately by sex within a latent variable SEM framework with illicit drug use (6 or more times in past year) as the latent factor.

**Results:**

More males initiated drug use (8.5%) than females (6.4%, *χ*2 (1) = 10.351, *p* = .001), but less so for Black males (AOR 0.33, 95% CI [0.18, 0.60]) and females (0.35 [0.23, 0.54]). Students initiating drug use more likely included students smoking cigarettes at baseline (males 1.40 [1.23, 1.59]; females 1.43 [1.24, 1.64]), using alcohol (males 1.04 [1.02, 1.06]; females 1.04 [1.02, 1.06]), or having cannabis using peers (males 1.79 [1.52, 2.11]; females 1.70 [1.49, 1.93]). Impulsivity domain associations differed by sex [negative urgency: females (1.23 [1.02, 1.49) and sensation seeking: males (1.33 [1.01, 1.75])]. History of unwanted/uncomfortable sexual experience predicted drug use for males (1.60 [1.09, 2.35]) and females (1.95 [1.45, 2.62]) but physical assault only for females (1.45 [1.08, 1.94]). Mood symptoms predicted drug use only for males [depression (0.73 [0.56, 0.95]); anxiety (1.40 [1.04, 1.89])].

**Conclusions:**

Risk factors for initiating drug use during college differ by sex. As substance use during early age predisposes one for addiction, sex‐ and gender‐informed interventions for young adults are needed.

## Graphical abstract

This study prospectively follows a large and diverse sample of students through the college years and is a useful addition to the literature on illicit drug use in a nontreatment seeking student population in the current opioid crisis. Among young adult students at the start of college, certain factors (i.e., tobacco or alcohol, peer drug use) were associated with subsequent initiation of occasional illicit drug use for both males and females while others differed across sex (i.e., mood symptoms, trauma histories), highlighting key areas to focus sex‐specific prevention efforts.

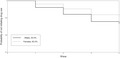

## 1 INTRODUCTION

Substance use disorders are generally more common among males than females across the life span, even though the size of this gender gap varies by substance and age (Evans‐Polce et al., 2015; Schulenberg et al., 2018; Woolf & Schoomaker, 2019). There is growing evidence suggesting sex and gender modify one's development and course of a substance use disorder (Di Nicola et al., 2017; Huhn et al., 2019; Kendler et al., 2015; McHugh et al., 2018; Polak et al., 2015). However, studies attempting to better elucidate sex and gender differences have been commonly met with mixed results. It has been hypothesized that these mixed findings are due to lack of prospective data collection, differences in methodology, populations, and substances studied (Greenfield et al., 2007; Ling et al., 2019; McHugh et al., 2018). Most importantly, few studies have stratified their data by sex to describe sex‐specific risks for addiction even though the National Institute on Drug Abuse (NIDA) has recommended that substance use research prioritize sex in design and analyses (Greenfield et al., 2011).

Further, the young adult years are known as stressful ones (Arnett, 2000) and comprise a critical period for long‐term health outcomes (Arnett, 2000). Notably, substance use during this time is associated with higher risk of subsequent addiction (Green et al., 2012), which carries with it a host of negative impacts on health and social functioning. The lifecourse perspective is useful to investigate this population, such as what subgroups are most likely to develop a substance use disorder and be affected by poor outcomes related to their substance use (Fothergill & Ensminger, 2006). The lifecourse model addresses the variation of protective and risk factors across developmental stages (e.g., distal and proximal factors) which contribute to health, underlying supportive and challenging mechanisms, the complexities between biopsychosocial factors, and how all these vary across time and between persons (Hser et al., 2007).

Sex is vital in a lifecourse framework, as it shapes one's life stages. Application of a sex‐informed lifecourse framework for substance use has led to identification of important sex differences over the life span among urban community‐based populations, highlighting new areas for investigation (Fothergill & Ensminger, 2006; Green et al., 2012). Multiple distal and proximal factors across individual to community levels of influence associated with substance use and addiction have been described in the literature, such as impulsivity (Moeller et al., 2001) and peer substance use (Grigsby et al., 2016). Further, some of these factors differ between males and females in their strengths of associations with substance use outcomes, such as family history, comorbid psychiatric disorders, and trauma (Kendler et al., 2015).

Therefore, utilizing a lifecourse framework stratified by sex to achieve a better understanding of sex‐specific distal and proximal risk profiles for substance use disorder among college males and females could help tailor targeted future sex/gender‐informed prevention and treatment strategies for this specific population. With a focus on drug use, the primary aim of the current study was to prospectively assess sex‐specific distal and proximal factors, measured during college entry, that would be associated with initiation of occasional illicit drug use during college years among a cohort of young adults. The secondary aim was to describe factors associated with initiation of illicit drug use before college entry stratified by sex. In this way, we aim to compare factors that impact the onset of occasional illicit drug use during college (primary aim) with factors that impact the onset of occasional use prior to college (secondary aim). Our study adds new findings to the field with its longitudinal design, sex‐stratified analyses and focus on illicit substance use initiation in a nontreatment seeking population contemporary to the opioid crisis.

## 2 MATERIALS AND METHODS

### 2.1 Participants and procedures

This study was a secondary data analysis from the Spit for Science (S4S) data registry, a prospective cohort study. S4S is an ongoing university‐wide research project located in the southeast United States, which longitudinally assesses genetic and environmental influences on substance use and psychiatric disorders in a representative majority of incoming freshman at a large urban university. The present study includes baseline (i.e., freshman year) as well as subsequent four waves of data (spring freshman, spring sophomore, spring junior, spring senior) from four cohorts of S4S (total *N* at baseline = 9.947). Approximately 2 weeks before arrival on campus, invitation to the online survey was mailed to all incoming freshmen aged 18 or older. Participants completed an online survey during the fall of their freshman year assessing a variety of factors including childhood experiences, personality, relationships, and behavior; they received $10 and a t‐shirt as compensation. After this, they were contacted in the spring of freshman year for follow‐up and then every year after this. Data collection for S4S began in the fall of 2011, and five cohorts of incoming freshman students have been enrolled in the study (incoming students in 2011–2014 and 2017). Detailed information concerning recruitment has been published elsewhere (Dick et al., 2014). Participants were representative of the broader student population in terms of both sex and race/ethnicity: 47% White, 22% Black/African American, 18% Asian, 6% Hispanic, 10% other race/ethnicity, majority female and mostly between the ages of 18–24 (In: Education USDO, editor, 2018). Self‐reported sex was assessed by asking participants about their sex (dichotomous: male–female). The participation rate across all 4 years of study was nearly 70% (Dick, 2017). The evaluation of attrition across time using logistic regression predicting completion status found that females were statistically significantly more likely than males to remain in the study (across all time points). Further, as compared to White participants, Black participants were more likely to complete (across all time points), Asian participants were more likely to complete at sophomore, junior, and senior year of college, and Hispanic participants were more likely to complete at junior and senior year (as compared to White participants). The university Institutional Review Board (IRB) approved all study procedures, and informed consent was obtained from all study participants. Study data were collected and managed using REDCap (Research Electronic Data Capture) electronic data capture tools (Harris et al., 2009).

### 2.2 Measures

#### 2.2.1 Primary aim

For analysis of our primary aim, participants were included if they did not endorse a history of illicit drug use before college entry. Illicit drug use was defined as use of cocaine, misuse of opioids or misuse of stimulants. Thus, participants were excluded from the primary aim analysis if they answered “yes” to the following question for any of these 3 substances at baseline: “*Have you used any of the following drugs for non‐medical use? Non‐medical use means on your own, without a doctor's prescription, in greater amounts than prescribed, or for reasons other than your doctor recommended*.”

##### Initiation of occasional illicit drug use

Participants were asked in the yearly follow‐up surveys “*Have you used any of the following drugs for non‐medical use? Non‐medical use means on your own, without a doctor's prescription, in greater amounts than prescribed, or for reasons other than your doctor recommended over the last 12 months.”* In cohorts 1 and 2, participants who reported incident drug use were asked whether they used the drugs 6 or more times during the 12 months. In cohorts 3 and 4, participants were asked to report the number of times they used these drugs in the past 12 months. For the current analysis, participants who reported cocaine use, misuse of opioids, or misuse of stimulants 6 or more times during the past 12 months were coded as initiating occasional illicit drug use (i.e., = 1) while those who did not report using any of the 3 substances at least 6 times were coded as not initiating occasional illicit drug use (or 0).

#### 2.2.2 Predictors

All the predictor variables were assessed at baseline, that is, Fall semester of freshman year. The lifecourse perspective served as the study's conceptual framework to guide selection of distal and proximal factors available in the dataset for this exploratory analysis. Factors were chosen for analysis based on a comprehensive literature review of individual (e.g., depression, alcohol use), interpersonal (e.g., peer cannabis use, family structure), and environmental‐level (e.g., trauma, stressful events) risk factors (NIMHD, 2017) for addiction, with a focus on those shown to demonstrate sex differences (largely when males and females are analyzed within one study sample) in substance use risk and patterns.

##### Age

Participants' self‐reported age.

##### Race

Self‐reported race. Given the low number of participants in some of the ethnic categories (American Indian, Hispanic/Latino, more than one race, Native Hawaiian/Pacific Islander, Unknown), they were collapsed into an “Other” category, leaving Asian, Black, and Other as dummy‐coded variables in the analysis (with White being the reference group).

##### Sensation seeking and Negative urgency

Selected impulsivity domains were measured by an abbreviated version of the *UPPS Impulsive Behavior Scale* (Whiteside & Lynam, 2001). This abbreviated version was created and provided to us by the authors of the original UPPS‐P scale, comprising five subscales—lack of perseverance, lack of premeditation, positive urgency, and sensation seeking, with 3 items per each subscale, measured on a 1–4 scale, ranging from 1 = disagree strongly to 4 = agree strongly. Two specific domains were chosen given their known associations with substance use: sensation seeking, the tendency to seek out novel or exciting experiences and willingness to take risks to do so (Evans‐Polce et al., 2018; Moshier et al., 2013; Vest et al., 2016), and negative urgency, the tendency to act rashly when experiencing strong negative affect (Kaiser et al., 2016; Smith & Cyders, 2016; Vest et al., 2016). These two subscales were created by averaging across their three respective items with higher numbers indicating higher sensation seeking or negative urgency. The reliabilities of these measures in the current study were *α* = 0.72 for negative urgency and *α* = 0.62 for sensation seeking.

##### Family structure

The family structure was self‐reported and recoded into a dichotomous variable (two‐parent household versus. single parent), where 1 = two‐parent family and 0 = other type of family structure.

##### Parental education

Computed as the mean of highest attained education of mother and father with 10 response options ranging from “never went to school” (0) to “professional training beyond a college or university” (9).

##### Parental history of drug use

A dichotomous variable asking participants whether their parents ever had problems with drugs (1 = yes, 0 = no).

##### Prior trauma

Indicated by four different types of trauma: physical assault, sexual assault, other unwanted or uncomfortable sexual experience, natural disaster or transportation accident, coded as 1 = past experience with this type of trauma, and 0 = no past experience.

##### Stressful events

Assessed as a sum of 12 dichotomous items (with responses “yes” or “no”), asking participants whether any of the potentially stressful events happened to them, including the following: broken steady relationships, separation from a loved one or close friend, serious illness or injury, getting burglarized or robbed, trouble with the police, laid off or fired from job, major financial problems, serious housing problems, serious difficulty at school, mother and father had a serious illness or injury, and someone close to you had a serious illness or injury. This measure was adapted from Kendler, Karkowski, and Prescott (Kendler et al., 1998).

##### Tobacco use

Assessed as the cumulative number of cigarettes smoked in lifetime at baseline with five options, ranging from “none” (1) to “more than 200” (5).

###### Current personal alcohol use

Indicated as approximate grams of ethanol consumed per month. This measure was created from two items: 1. Participants' answer on an item asking about frequency of alcohol use in the past month (“never,” “monthly or less,” “2 to 4 times a month,” “2 to 3 times a week,” and “4 or more times a week.”); 2. Participants' answer on an item asking the number of drinks they had on a typical day when drinking, using the following categories: “1 or 2,” “3 or 4,” “5 or 6,” “7, 8, or 9,” and “10 or more.” The responses of frequency of alcohol use and number of drinks on a typical day drinking were converted to the midpoints for each response category. Then, the number of days participants were drinking was multiplied by the number of drinks per occasion, and this was multiplied by 14, reflecting 14 grams of pure alcohol, which is roughly the amount of alcohol included in a standard drink (Salvatore et al., 2016).

###### Number of peers using cannabis

Indicated by a single question with five response options (1 = “none,” 2 = “a few,” 3 = “some,” 4 = “most,” 5 = “all”), asking participants how many of their peers smoke cannabis.

###### Depression and anxiety symptoms

Measured using a subset of items (four items for each construct) from the SCL‐90 (Derogatis et al., 1973), with higher scores reflecting higher levels of depression or anxiety symptoms. The reliability of the depression symptoms scale was *α* = 0.80 and *α* = 0.82 for anxiety.

#### 2.2.3 Secondary aim

For the secondary aim, all participants were included in the study sample. The outcome assessed was occasional use of illicit drugs (use of cocaine, misuse of opioids, misuse of stimulants) before college entry, again dichotomized into “*used 6 or more times*” (1 = yes) and used 5 times or less (0 = no). The same distal and proximal factors were assessed as for the primary objective's analysis according to their baseline measurements aside from peer cannabis use, which for this analysis referred to one's high school (rather than college) peers.

### 2.3 Plan of analysis

Given the longitudinal nature of the data (baseline + four waves), our goal was to model the initiation of occasional illicit drug use (across four waves) among those college students with no previous drug use (as indicated at the baseline) separately by sex. To do this, we selected only those students who did not endorse a history of illicit drug use before college. Given the dichotomous nature of our outcome, we used a discrete time survival analysis. This was modeled within a structural equation modeling framework where the four waves are modeled as four indicators of a latent factor (illicit drug use), with equal loadings and zero residual variance of the latent factor. Then, the predictors are regressed on this latent factor. Individuals that initiate drug use change their status from “0” to “1” and are censored in subsequent waves. The survival rate then reflects individuals who did not initiate illicit drug use throughout 4 years of college. For our secondary aim, this was modeled within a logistic regression framework with lifetime use of illicit drugs as the outcome. To deal with missing data, we used multiple imputation with 100 imputed datasets. Given that individuals with missing data in survival analysis are censored on the subsequent time points (given the uncertainty about their status), we only imputed predictors and not the outcome variables. For the logistic regression, we imputed both predictors as well as the outcome variables. All analyses were stratified by sex. The predictor effects that were found to be statistically significant for either males or females were then directly compared by constraining their parameters to equality and using Wald test to assess the statistical significance of the model change. All analyses were done in Mplus 8.0 (Muthén & Muthén, 1998−2017).

## 3 RESULTS

**FIGURE 1:**
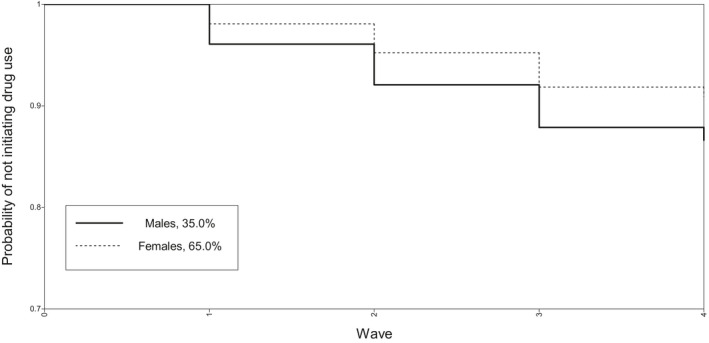
Survival rate (not initiating occasional drug use) across 4 years of college, segregated by sex. Among college students without a history of illicit drug use before college, the proportion of those not initiating occasional illicit drug use (*y*‐axis—survival rate) is shown. Overall, more males than females initiated occasional illicit drug use during college and earlier during the 4‐year (*x*–axis—“Wave”) study time frame

A total of 8,071 participants in S4S cohorts 1–4 were included in the primary objective's analysis. These included those who did not endorse a history of illicit drug use before college entry (1,279 participants [12.8%] reported ever using at least one of the substances in their lifetime). Also, these participants did not have missing data on prior illicit drug use (586 participants had missing values on prior use before baseline for the three drugs). Similar to the university's population, this included 5,120 (63.4%) women and 2,951 (36.6%) men. Over the 4 years of follow‐up, 484 participants (7.2%) initiated occasional illicit drug use (use of cocaine, misuse of opioids, misuse of stimulants). More males than females initiated occasional illicit drug use (8.5% men versus 6.4% women, *χ* ^2^ (1) = 10.351, *p* = .001). The Kaplan–Meier curve segregated by sex is shown in Figure 1. Descriptive statistics of the study population are outlined in Table 1.

**Table 1:** Descriptive statistics of study variables

	Males			Females		
	*M*/%	*SD*	Min/max	*M*/%	*SD*	Min/max
Asian	18.21%	0.39	0/1	15.61%	0.36	0/1
Black	14.45%	0.35	0/1	22.31%	0.42	0/1
Other	13.67%	0.34	0/1	14.27%	0.35	0/1
White	53.67%	0.33	0/1	47.81%	0.32	0/1
Age	18.65	0.67	18.02/32.07	18.56	0.55	18.01/31.4
Sensation seeking	3.08	0.65	1/4	2.85	0.70	1/4
Negative urgency	2.15	0.74	1/4	2.20	0.78	1/4
Two‐parent family	70.16%	0.46	0/1	64.24%	0.48	0/1
Parental education	7.12	1.94	0/9	6.95	1.98	0/9
Maternal drug use	5.00%	0.23	0/1	6.00%	0.23	0/1
Paternal drug use	12.00%	0.33	0/1	15.00%	0.35	0/1
History of sexual assault	7.00%	0.25	0/1	17.00%	0.38	0/1
Other prior unwanted or uncomfortable sexual experience	18.00%	0.39	0/1	42.00%	0.49	0/1
History of natural disaster or transportation accident	79.19%	0.41	0/1	80.58%	0.40	0/1
History of physical assault	35.00%	0.48	0/1	27.00%	0.44	0/1
History of stressful life events	4.22	5.12	0/24	5.25	5.65	0/24
Peer cannabis use in college	2.77	1.24	1/5	2.57	1.19	1/5
Peer cannabis use in high school	2.85	1.26	1/5	2.53	1.21	1/5
Depressive symptoms	2.02	0.90	1/5	2.29	0.93	1/5
Anxiety symptoms	1.56	0.72	1/5	1.78	0.81	1/5
Cumulative number of cigarettes smoked at baseline	1.90	1.30	1/5	1.62	1.06	1/5
Grams of alcohol per month	219.57	506.63	0/5108.18	126.82	322.77	0/5108.18

The results from survival analysis are listed in Table 2. For both males and females, Black participants were less likely to initiate occasional illicit drug use during college as compared to White participants (males AOR 0.33, 95%CI [0.18, 0.60]; females 0.35 [0.23, 0.54]). Among females, those that were grouped as “Other” ethnic category also were less likely (0.58 [0.39, 0.86]). Across sex, certain substance use‐related factors were associated with higher risk of initiating occasional illicit drug use: cumulative cigarette use as of baseline (men 1.40 [1.23, 1.59]; females 1.43 [1.24, 1.64]), using alcohol (males 1.04 [1.02, 1.06]; females 1.04 [1.02, 1.06]), or having peers who use cannabis (males 1.79 [1.52, 2.11]; females 1.70 [1.49, 1.93]). Regarding impulsivity dimensions, negative urgency was positively associated with illicit drug use initiation only in females (1.23 [1.02, 1.49), while sensation seeking was associated with illicit drug use among males only (1.33 [1.01, 1.75]). History of unwanted or uncomfortable sexual experience was a significant positive predictor for both males (1.60 [1.09, 2.35]) and females (1.95 [1.45, 2.62]). Other significant distal and proximal factors differed between males and females. For females only, history of physical assault was a significant predictor of illicit drug initiation (1.45 [1.08, 1.94]). Males with depressive symptoms were less likely to initiate drug use (0.73 [0.56, 0.95]), while higher levels of anxiety were associated with higher likelihood of drug use initiation (1.40 [1.04, 1.89]), again only among males.

**Table 2:** Distal and proximal factors associated with initiation of occasional illicit drug during college

	Males						Females					
	*B*	*SE*	*p*	OR	95% OR CI		*B*	*SE*	*p*	OR	95% OR CI	
Asian^a^	−0.20	0.25	.419	0.82	0.50	1.34	−0.36	0.20	.072	0.70	0.47	1.03
Black^a^	**−1.12**	**0.31**	**<.001**	**0.33**	0.18	0.60	**−1.04**	**0.22**	**<.001**	**0.35**	0.23	0.54
Other^a^	−0.06	0.24	.788	0.94	0.59	1.49	**−0.55**	**0.20**	**.007**	**0.58**	0.39	0.86
Age	−0.09	0.12	.460	0.92	0.73	1.15	−0.28	0.18	.120	0.75	0.53	1.08
Sensation seeking	**0.28**	**0.14**	**.044**	**1.33**	1.01	1.75	0.06	0.11	.611	1.06	0.85	1.32
Negative urgency	0.10	0.12	.372	1.11	0.88	1.39	**0.21**	**0.10**	**.029**	**1.23**	1.02	1.49
Two‐parent family	−0.32	0.18	.086	0.73	0.51	1.05	0.10	0.16	.509	1.11	0.82	1.50
Parental education	0.07	0.05	.133	1.07	0.98	1.18	0.03	0.04	.480	1.03	0.95	1.10
Maternal drug use	−0.11	0.34	.743	0.89	0.46	1.75	0.08	0.29	.780	1.08	0.62	1.90
Paternal drug use	−0.12	0.28	.662	0.89	0.52	1.52	0.00	0.21	.998	1.00	0.67	1.50
History of sexual assault	0.35	0.31	.251	1.42	0.78	2.58	−0.01	0.18	.953	0.99	0.70	1.41
Other prior unwanted or uncomfortable sexual experience	**0.47**	**0.20**	**.016**	**1.60**	1.09	2.35	**0.67**	**0.15**	**<.001**	**1.95**	1.45	2.62
History of natural disaster or transportation accident	0.04	0.23	.848	1.04	0.67	1.62	−0.24	0.18	.186	0.79	0.56	1.12
History of physical assault	0.29	0.18	.104	1.33	0.94	1.89	**0.37**	**0.15**	**.014**	**1.45**	1.08	1.94
History of stressful life events	0.00	0.01	.782	1.00	0.98	1.03	−0.01	0.01	.315	0.99	0.97	1.01
Peer cannabis use in college	**0.58**	**0.08**	**<.001**	**1.79**	1.52	2.11	**0.53**	**0.07**	**<.001**	**1.70**	1.49	1.93
Depressive symptoms*	**−0.32**	**0.14**	**.020**	**0.73**	0.56	0.95	0.06	0.10	.562	1.06	0.87	1.29
Anxiety symptoms*	**0.34**	**0.15**	**.024**	**1.40**	1.04	1.89	−0.07	0.11	.498	0.93	0.75	1.15
Cumulative cigarettes smoked	**0.34**	**0.07**	**<.001**	**1.40**	1.23	1.59	**0.36**	**0.07**	**<.001**	**1.43**	1.24	1.64
Drinks per week	**0.04**	**0.01**	**<.001**	**1.04**	1.02	1.06	**0.04**	**0.01**	**<.001**	**1.04**	1.02	1.06

Note

Bold are predictors significant at *p* < .05.

^a^ The reference group is White.

* The predictors that were identified to be statistically significant in at least one group (in bold) were directly compared to test whether the effect varied by sex. Those that showed statistically significant difference (*p* < .05) are indicated by *.

Directly comparing the estimates that were significant in at least one group to assess whether the effects were moderated by sex, we found that the effect of depressive symptoms significantly differed by sex such that higher levels of depressive symptoms among males was predictive of lower likelihood of initiating drug use, while the effect was nonsignificant for females. Furthermore, higher levels of anxiety symptoms among males predicted higher likelihood of initiating drug use, but this was again statistically different when compared to females, for whom the effect was nonsignificant.

For the secondary aim, 14.4% of participants used illicit drugs 6 + times before college entry, including 296 (4.9%) females and 276 (7.3%) males. The results from the logistic regression are shown in Table 3. White participants were most likely to have used illicit drugs at least 6 times before college [Asian (males AOR 0.47, 95% CI [0.25, 0.86] and females 0.42 [0.23, 0.75]); Black males (0.52 [0.28, 0.99]) and females (0.24 [0.13, 0.46])]. Impulsivity domains were associated with prior drug use for females only [sensation seeking (1.31 [1.03, 1.66]) and negative urgency (1.26, [1.03, 1.55])]. Similarly, females with higher levels of depressive symptoms were more likely to have prior illicit drug use (1.24, [1.02, 1.51]). Proximal factors associated with illicit drug use history for both sexes included cumulative cigarette use as of baseline (males 1.84 [1.65, 2.06]; females 1.87 [1.67, 2.09]), alcohol use (males 1.03 [1.02, 1.05]; females 1.02 [1.01, 1.04]), and high school peer cannabis use (males 1.62 [1.39, 1.90]; females 1.69 [1.46, 1.96]). Comparing the effects of significant predictors by sex, we did not find any of the identified predictors to significantly differ between males and females.

**Table 3:** Distal and proximal factors associated with history of occasional illicit drug before college entry

	Males						Females					
	*B*	*SE*	*p*	OR	95% OR CI		*B*	*SE*	*p*	OR	95% OR CI	
Asian^a^	**−0.76**	**0.31**	**.015**	**0.47**	0.25	0.86	**−0.88**	**0.30**	**0.004**	**0.42**	0.23	0.75
Black^a^	**−0.65**	**0.32**	**.045**	**0.52**	0.28	0.99	**−1.42**	**0.33**	**<0.001**	**0.24**	0.13	0.46
Other^a^	−0.32	0.22	.149	0.73	0.47	1.12	−0.23	0.18	0.207	0.79	0.55	1.14
Age	−0.04	0.08	.601	0.96	0.81	1.13	−0.02	0.14	0.885	0.98	0.75	1.28
Sensation seeking	0.10	0.14	.463	1.11	0.85	1.45	**0.27**	**0.12**	**0.024**	**1.31**	1.03	1.66
Negative urgency	0.10	0.11	.363	1.11	0.89	1.38	**0.23**	**0.11**	**0.027**	**1.26**	1.03	1.55
Two‐parent family	−0.01	0.17	.949	0.99	0.71	1.37	−0.02	0.15	0.909	0.98	0.73	1.32
Parental education	0.06	0.05	.188	1.06	0.97	1.16	0.04	0.04	0.294	1.04	0.96	1.13
Maternal drug use	0.35	0.30	.235	1.42	0.80	2.55	0.12	0.25	0.621	1.13	0.69	1.85
Paternal drug use	0.08	0.22	.714	1.09	0.70	1.68	−0.01	0.19	0.977	0.99	0.69	1.44
History of sexual assault	0.05	0.30	.869	1.05	0.59	1.88	0.05	0.18	0.793	1.05	0.73	1.50
Other prior unwanted or uncomfortable sexual experience	0.31	0.19	.104	1.36	0.94	1.98	0.28	0.17	0.105	1.32	0.94	1.84
History of natural disaster or transportation accident	−0.01	0.21	.976	0.99	0.66	1.49	0.19	0.20	0.337	1.21	0.82	1.78
History of physical assault	0.22	0.16	.168	1.25	0.91	1.72	0.26	0.16	0.108	1.30	0.94	1.79
History of stressful life events	0.01	0.02	.566	1.01	0.98	1.04	0.01	0.01	0.731	1.01	0.98	1.03
Peer cannabis use in high school	**0.49**	**0.08**	**<.001**	**1.62**	1.39	1.90	**0.53**	**0.08**	**<0.001**	**1.69**	1.46	1.96
Depressive symptoms	0.19	0.11	.087	1.21	0.97	1.49	**0.22**	**0.10**	**0.028**	**1.24**	1.02	1.51
Anxiety symptoms	−0.06	0.13	.655	0.94	0.73	1.22	−0.02	0.10	0.851	0.98	0.80	1.20
Cumulative cigarettes smoked	**0.61**	**0.06**	**<.001**	**1.84**	1.65	2.06	**0.63**	**0.06**	**<0.001**	**1.87**	1.67	2.09
Drinks per week	**0.03**	**0.01**	**<.001**	**1.03**	1.02	1.05	**0.02**	**0.01**	**0.030**	**1.02**	1.01	1.04

Note

Bold are predictors significant at *p* < .05.

^a^ The reference group is White.

* The predictors that were identified to be statistically significant in at least one group (in bold) were directly compared to test whether the effect varied by sex. None showed statistically significant differences (*p* < .05).

The simplified comparison of occasional illicit drug use during and before college is shown in Table 4.

**Table 4:** Overview of distal and proximal factors associated with occasional illicit drug use

	Initiation of occasional illicit drug use during college		Occasional illicit drug use before college	
	Males	Females	Males	Females
Overall factors				
Asian race	↔	↔	↓	↓
Black race	↓	↓	↓	↓
Other nonwhite race	↔	↓	↔	↔
Sensation seeking	↑	↔	↔	↑
Negative urgency	↔	↑	↔	↑
Distal factors				
History of unwanted or uncomfortable sexual experience	↑	↑	↔	↔
History of physical assault	↔	↑	↔	↔
Proximal factors				
Peer cannabis use	↑	↑	↑	↑
Depressive symptoms	↓*	↔*	↔	↑
Anxiety symptoms	↑*	↔*	↔	↔
Cumulative cigarettes smoked as of baseline	↑	↑	↑	↑
Number of alcoholic drinks per week	↑	↑	↑	↑

Note

↑ reflects positive effect, ↓ reflects negative effect, and ↔ reflects no significant effect.

* The predictors that were identified to be statistically significant in at least one group were directly compared to test whether the effect varied by sex. Those that showed statistically significant difference (*p* < .05) are indicated by *.

## 4 DISCUSSION

The current study prospectively explored the effect of distal and proximal factors on the initiation of occasional illicit drug use (defined as using more than six times in the past year) across 4 years of college among students without a precollege history of drug use, separately for males and females. Among young adult students at the start of college, certain factors (i.e., tobacco or alcohol, peer drug use) were associated with subsequent initiation of occasional illicit drug use for both males and females while others were not consistent across sex (i.e., mood symptoms), highlighting key areas to focus sex‐specific prevention efforts. Further, these protective and risk factors were distinct from those associated with initiation of illicit drug use before college entry, highlighting the importance of incorporating a lifecourse framework into the study of sex differences in addiction.

For both males and females, personal alcohol use and tobacco use were consistent proximal factors associated with illicit drug use both among individuals who initiated occasional use before college entry and during college. Notably, self‐reported parental substance use was not a risk factor, but having peers who use cannabis was a risk factor at both time points. The implications of the consistency in these associations across time are twofold. First, there may be certain underlying dispositions persisting across the lifecourse that heighten the risk for substance use and addiction (Fothergill & Ensminger, 2006; Grigsby et al., 2016), highlighting areas for potential interventions. Second, focusing on reducing tobacco and alcohol use in adolescents and young adults could serve to also prevent subsequent illicit drug use. Fortunately for these purposes, in the college environment, tobacco and alcohol use are not taboo topics, allowing targeted public health prevention efforts to easily reach the population using these substances more easily than in other contexts. Lastly, our findings emphasize the importance community‐based interventions for this population given that we found peer substance use to predict drug use while parental substance use did not, a different finding than noted in prior literature (Kendler et al., 2015).

Previous work links impulsivity domains to illicit drug use (Moeller, Dougherty, et al., 2001). Our study prospectively examined the role of impulsivity on illicit substance use in a nontreatment seeking population. Additionally, our sex‐stratified results support recent work highlighting varying effects of specific impulsivity domains (e.g., urgency, sensation seeking) on substance use (Kaiser et al., 2016; Vest et al., 2016) as well as the need to study such associations through a sex/gender lens (Evans‐Polce et al., 2018). Urgency has been linked with alcohol problems among young adults in other prospective studies (Kaiser et al., 2016; Stojek & Fischer, 2013), and our results add to this literature with negative urgency's association with subsequent illicit drug use. The role of affect in urgency makes it unique within the multidimensional impulsivity framework, especially when considering how to tailor personality specific interventions to populations of interest [e.g., the need to target both the impulsive trait itself and sources of negative affect; (Bold et al., 2017; Kozak et al., 2018)].

Considering the roles of sex and gender, prior work has found either no difference in the impulsivity to substance use relationship by sex (Di Nicola et al., 2017) or mixed results. For example, Monitoring the Future data has found sensation seeking to be associated with tobacco, alcohol and cannabis use among adolescents and young adults, but the strength and direction of these associations varied across time and by sex (Evans‐Polce et al., 2018). Although we did not find the effects of these associations to significantly differ by sex, our sex‐stratified analyses suggest that variations by sex and time (use before college or initiation during college) in the impacts of impulsivity domains on the risk of illicit drug use remain salient for males and females. Overall, our findings further support employing a sex‐specific lifecourse framework in the study of substance use and addiction. Neuroscience‐informed psychoeducation interventions are emerging as effective strategies that can be tailored to an individual's specific needs (Ekhtiari et al., 2017), such as compromised impulsivity domains, to prevent and treat substance use disorders (Vassileva & Conrod, 2019). Our study prospectively delineates sex‐specific roles of impulsivity in the development of illicit drug use among young adults, highlighting examples of how such interventions can be individualized for a specific population. Overall, our data support the need for more work describing links between neuroscience domains and substance use across development by sex/gender in order to best tailor interventions for young males and females and reduce risk of subsequent addiction.

Trauma history is a well‐known factor associated with substance use and addiction (Kilpatrick et al., 2003). By stratifying analyses by sex at both time points, our study elucidated this relationship further. Sexual assault and other unwanted/uncomfortable sexual experience history increased odds of initiating occasional illicit drug use during college for both males and females. Additionally, females with a prior physical assault were also more likely to initiate occasional drug use during college, although the direct comparison of the effect of this association by sex did not indicate statistical significance. Our results support previous work highlighting sexual harassment as a risk factor for alcohol use among females but not males (Freels et al., 2005). As for males, previous studies on the influence of sexual assault on subsequent drug use outcomes have had mixed results (Rougemont‐Bucking et al., 2017, 2018), an area in need of further investigation. Nonetheless, our findings support further strengthening programs targeting violence and harassment in the college environments, especially given the current #MeToo climate, as doing so may also reduce illicit drug use initiation for both young males and females. Also, our findings of prior trauma being associated with initiation of drug use during subsequent young adult years but not use prior to college entry indicate the importance of utilizing a lifecourse framework to better understand substance use, as it appears adolescence is a critical period with regard to the elevated risk of subsequent substance use related to a traumatic experience.

Prior work focused on sex‐specific associations between mood and substance use using longitudinal data has generally found stronger associations for females (McHugh et al., 2018). Using a lifecourse perspective, we provide further detail to this association as depressed females were slightly more likely to initiate illicit drug use at an early age before college entry without a significant association during college, although this difference in effect by sex did not reach statistical significance. For males only, depressive symptoms were a protective factor for occasional illicit drug use initiation during college, and interestingly anxiety was a risk factor. This effect was then found to significantly differ between males and females, providing further support for the moderating effect of sex on the relationship between engagement in illicit drug use during college and depressive or anxiety symptoms. In the college environment, males with depressive symptoms may be less likely to interact with peers, such as at parties, leading to less drug use. The association between anxiety symptoms and heightened risk of occasional illicit drug use in males warrants further investigation.

The strengths of our study lie first in its longitudinal design, enabling us to capture the dynamic development of occasional illicit drug use throughout college years among students with no precollege drug experience. This prospective design allowed us to elucidate predictors of substance use by sex with more certainty than prior retrospective or cross‐sectional studies have done (Di Nicola et al., 2017), adding clarity to existing conundrums revolving around questions like “what came first? – substance use or this risk factor?”. Second, given its large and ethnically heterogeneous sample size, this study provides robust evidence for salient predictors specific to illicit drug use among college students. Furthermore, segregating the analyses by sex emphasizes the divergent pathways through which males and females develop occasional drug use.

Our study also has limitations. First, all our predictor and outcome variables are solely from self‐report. This could result in misclassification or social desirability bias, especially for topics with more stigma attached to them such as drug use (either personal or reported parental use). Notably, we did find our prevalence of illicit drug use initiation in our study cohort to be consistent with findings from national data (Schulenberg et al., 2018). Another limitation is the lower internal consistency of the abbreviated UPPS‐P sensation seeking measure found in this dataset, which might have negatively affected the predictive power of this construct.

Also, sex was assessed as a binary variable without additional items assessing gender identity; further work should be replicated using comprehensive variables for both sex and gender. Our data are based on a convenience sample of students at a single educational institution. Also, some students matriculated almost 10 years ago. Substance use trends fluctuate over time across age groups; thus, it is important that work at this intersection continues in order to inform interventions applicable to the current populations at risk. Finally, the sample was less balanced in terms of sex ratio with a majority of female respondents. Even though there was still an adequate number of men, future studies should strive to have more balanced samples especially as males are more likely to engage in drug use (McCabe et al., 2007) as well as drop out of college (Buchmann & Diprete, 2006), further limiting their sample size in follow‐up data collections.

As substance use during early age disposes one for subsequent addiction, related interventions for young adults are needed and should consider sex and gender while identifying vulnerable subgroups to target. Overall, our findings provide guidance public health officials can use to tailor substance use prevention and harm reduction efforts for college students using a sex‐informed approach, such as strengthening substance use community support programs with peers, prioritizing both licit (alcohol, tobacco) and illicit substance use in funding applications, building more robust trauma programs for males and females as well as considering implementation of emerging neuroscience based education strategies on substance use disorders for college students.

## CONFLICT OF INTEREST

None declared.

## AUTHOR CONTRIBUTIONS

DD oversaw all aspects of the parent study. CEM conceptualized the research question for this secondary analysis with assistance from AJK and oversight by DD and FGM. AJK performed all data analysis and assisted with data interpretation as a collaborative effort from DD and FGM, oversaw by CEM. CEM and AJK wrote the first draft of the manuscript. All authors assisted in manuscript revisions and approved the final draft of the manuscript for publication.

### Peer Review

The peer review history for this article is available at https://publons.com/publon/10.1002/brb3.1959.

## Data Availability

The data that support the findings of this study are openly available to qualified researchers via dbGaP (phs001754) at https://www.ncbi.nlm.nih.gov/projects/gap/cgi‐bin/study.cgi?study_id=phs001754.v2.p1
